# Effects of chronic dexamethasone administration on hyperglycemia and insulin release in goats

**DOI:** 10.1186/s40104-018-0242-4

**Published:** 2018-03-16

**Authors:** Liqiong Niu, Qu Chen, Canfeng Hua, Yali Geng, Liuping Cai, Shiyu Tao, Yingdong Ni, Ruqian Zhao

**Affiliations:** Key Laboratory of Animal Physiology & Biochemistry, Nanjing Agricultural University, Nanjing, 210095 People’s Republic of China

**Keywords:** Blood glucose, Dexamethasone, Gluconeogenesis, Goat, Liver

## Abstract

**Background:**

Dexamethasone (Dex), a synthetic glucocorticoid, is among the most commonly used drugs worldwide in animals and humans as an anti-inflammatory and immunosuppressive agent. GC has profound effects on plasma glucose level and other metabolic conditions. However, the effect of prolonged use of Dex on glucose metabolism in ruminants is still unclear.

**Results:**

Ten goats were randomly assigned to two groups: the control goats were injected with saline, and the Dex-treated goats were intramuscularly injected daily for 21 d with 0.2 mg/kg Dex. The results showed that plasma glucose and insulin concentrations were significantly increased after Dex administration (*P* < 0.05). Additionally, the content of hepatic glycogen was also markedly increased in Dex-treated goats (*P* < 0.01), while the content of glycogen in dorsal longissimus was unchanged by Dex (*P* > 0.05). The expression of several key genes, involved in blood glucose regulation, was detected by real-time PCR in the small intestine, skeletal muscle and liver. The expression of glucose transporter type 2 (*GLUT2*), sodium-glucose transporter 1 (*SGLT1*) and sodium-potassium ATPase (*Na-K/ATPase*) in the small intestine were generally increased by Dex, and *GLUT2* mRNA expression was significantly up-regulated (*P* < 0.05). In liver, the expression of genes involved in gluconeogenesis including glucose-6-phosphatase catalytic subunit (*G6PC*), cytosolic form of phosphoenolpyruvate carboxykinase (*PCK1*) and pyruvate carboxylase (*PC*), were significantly down-regulated by Dex. However, the protein expression levels of PCK1 & PCK2 were significantly increased by Dex, suggesting a post-transcriptional regulation. In dorsal longissimus, the mRNA expression of genes associated with gluconeogenesis and the insulin signaling pathway were generally up-regulated by Dex, but the mRNA expression of two markers of muscle atrophy, namely F-box protein 32 (*FBXO32/Atrogin1*) and muscle RING-finger protein 1 (*MuRF1*), was not altered by Dex.

**Conclusions:**

Taken together, these results indicate that chronic administration of a low dosage of Dex induces hyperglycemia mainly through gluconeogenesis activation in the goat liver.

## Background

The hypothalamic-pituitary-adrenal (HPA) axis plays an important role in the homeostatic maintenance of blood glucose under normal or stress conditions [1]. Glucocorticoids (GCs), the end-hormones of the HPA axis, play a vital role in regulating glucose homeostasis [2]. Dexamethasone (Dex), a synthetic GC drug, is widely used as an anti-inflammatory and anti-allergic agent to treat various inflammatory, allergic and autoimmune diseases [3, 4]. However, high doses or chronic use of GCs can produce undesired side effects, such as central obesity, hepatic hyperlipidemia, hypertension, glucose intolerance and muscular atrophy [5, 6]. Accordingly, these adverse effects to the host often limit the utility of chronic GC use.

As in non-ruminant animals, glucose is an important metabolic nutrient metabolism in ruminants, and also a primary precursor in the synthesis of lactose, which determines milk yield [7]. Ruminants normally absorb only small amounts of dietary glucose in the small intestine due to the extensive fermentation of carbohydrates in the forestomachs [8]. As a result, ruminants primarily depend on hepatic gluconeogenesis as their source of glucose to meet their energy demand, utilizing precursors like propionate and glucogenic amino acids [9]. Chronic treatment with GC drugs, such as Dex, has been associated with hyperglycemia and insulinemia in both animal and human studies [10, 11]. These undesirable GC-induced effects have been focused on the liver, skeletal muscle and adipose tissue. The studies show that GCs induce peripheral insulin resistance, in vivo and in vitro [10–12], by increasing hepatic glucose output and decreasing the peripheral glucose uptake. However, the effects of GC-induced insulinemia are still contentious. For instance, after GC exposure, blood insulin concentration has been found to be increased [13], unaltered [14] or decreased in mammals [15]. It has been reported that Dex reduces insulin secretion by pancreatic β cells due to the oxidative stress on these cells, and β cells have been found to be particularly vulnerable and susceptible to reactive oxygen species (ROS) toxicity [16]. In cows, a single Dex injection induced a sharp increase in blood glucose concentration and then quickly returned to normal level [17]. The milk-reducing effect of Dex in cows is well known [18] and is related to its ability to decrease glucose uptake by skeletal muscle, adipose tissue as well as the mammary gland, which leads to an insulin resistance state [11, 18]. However, to date, data about the effects of chronic exposure to Dex on glycometabolism are scarce in ruminants. Accordingly, this research focused on evaluating glycometabolic effects on an experimental model of chronic exposure to GC in goats.

## Methods

### Animals and experimental procedures

Ten healthy male goats, 25 ± 1.0 kg in body weight, were raised in individual stalls in a conventional animal feeding housing facility at Nanjing Agricultural University (Nanjing, China). Body weight was measured at 1, 7, 14 and 21 d before the beginning of Dex administration, and food intake was measured daily during the experiment. The animals were fed twice daily at 08:00 and 18:00 h, and had free access to water during the experimental period. All goats received a high-grain diet composed of 43% corn, 5% wheat bran, 17% mixed concentrate and 35% forage. Animals were adapted to all procedures of sampling and treatment before treatments. The dose of Dex was determined based on a previous study by Emikpe et al. [19]. The ten goats were randomly assigned to two groups: the control (Con) group was intramuscularly injected with saline, and the Dex-treated group was intramuscularly injected with Dex 0.2 mg/kg at 07:30 h. before morning feeding for 21 d.

### Sample collection

Plasma samples were obtained on d 1, 7, 14 and 21 of the experiment shortly before the injection and morning feeding from the jugular vein. Samples of the duodenum, dorsal longissimus muscle and liver tissue were collected at the end of the experiment. After an overnight fast, all goats were weighed and killed by an injection of xylazine (0.5 mg/kg of body weight; Xylosol; Ogris Pharma, Wels, Austria) and pentobarbital (50 mg/kg of body weight; Release; WDT, Garbsen, Germany). Immediately after slaughtering, the duodenum, dorsal longissimus muscle and liver tissue samples were collected and frozen immediately in liquid nitrogen and stored at − 80 °C for subsequent extraction of RNA and proteins.

### Measurement of plasma parameters

Plasma glucose was measured using an automatic biochemical analyzer (7020, Hitachi, Tokyo, Japan), following the manufacturer’s instructions. Plasma insulin was determined using a RIA insulin kit (Beijing North Institute of Biological Tec., Beijing, China), following the manufacturer’s instructions. The sensitivity of the assay was 1 μIU/mL, the intra-assay coefficient of variation (CV) was 6.9%, and all samples were measured in the same assay to avoid interassay variability. Plasma cortisol was determined using a RIA cortisol kit (Beijing North Institute of Biological Tec.), following the manufacturer’s instructions. The sensitivity of the assay was 1 ng/mL, the intra-assay CV was 7.0%, and all samples were measured in the same assay to avoid interassay variability. Glycogen was measured using a Glycogen kit (Jiancheng Co. Ltd., Nanjing, China), following the manufacturer’s instructions. Glucagon was determined with a Glucagon ELISA kit (Feiya Co. Ltd., Nanjing, China), following the manufacturer’s instructions. The sensitivity of the assay was 1 μIU/mL, the intra-assay CV was 6.5%, and all samples were measured in the same assay to avoid interassay variability.

### Staining for PAS

The isolated dorsal longissimus muscle and liver tissue samples were washed three times with normal saline and fxed with a 10% formalin solution for 48 h at 4 °C, dehydrated with ethanol, and embedded with paraffin. The paraffn-embedded slices were deparaffinized, hydrated with distilled water and oxidized for 10 min at 40 °C in a 0.5% periodic acid solution. The sections were then washed with distilled water, immersed in Schiff reagent, washed in distilled water, counterstained with Mayer’s hematoxylin, washed in distilled water, dehydrated in a gradient of ethanol and ultimated sealed using a synthetic mounting medium. The slices of each staining were all were viewed under a light microscope (DM3000 LED; Leica, Wetzlar, Germany).

### RNA isolation, cDNA synthesis and real-time PCR

Total RNA was extracted from each tissue samples using the TRIzol reagent (15596026; Invitrogen, Shanghai, China). The concentration and quality of the RNA were assessed with a NanoDrop ND-1000 Spectrophotometer (Thermo Fisher Scientific, Waltham, MA, USA). Then 2 μg of total RNA were treated with RNase-Free DNase (M6101; Promega, Madison, WI, USA) and reverse transcribed according to manufacturer’s instructions. Real-time PCR was performed in an Mx3000P qPCR system (Agilent Technologies, Santa Clara, CA, USA) using the Stratagene Mx3000P qPCR kit (Stratagene, La Jolla, CA, USA). The 18S RNA, which is not affected by the experimental factors, was chosen as the reference gene. In order to control the PCR efficiency, two microliters of diluted cDNA (1:40, *v/v*) was used for real-time PCR. The PCR protocols were as follows: initial denaturation (1 min at 95 °C), then a three-step amplification stage (20 s at 95 °C, 20–30 s at 62 °C, 30 s at 72 °C) was repeated 45 times. All the primers used are listed in Table 1, and were synthesized by the Tsingke Company (Nanjing, China). The qPCR products were all directly sequenced. Additionally, melt curves were checked to confirm the specificity of the primers. The method of 2^- △△Ct^ was used to analyze the real-time PCR data, and gene mRNA levels were expressed as the fold change relative to the mean value of the control group.

**Table 1:** PCR primers for glucose metabolism genes

Gene	Sequence 5′→3’	GenBank accession no.	Product length, bp
*GLUT1*	Forward: AACCGCAACGAGGAGAACC	NM_001314223.1	155
	Reverse: TGCAGCACCACGGCAAT		
*GLUT2*	Forward: GAGGCATATCAGGACTCTAC	XM_005675321.3	156
	Reverse: AGGGCACCAATAGCAC		
*SGLT1*	Forward: CACCCATCGCAGCAGT	XM_018060890.1	199
	Reverse: CGGGCGTCTTGAATGT		
*Na-K/ATPase*	Forward: CCTCGAAATCCATTGCTTATACC	[38]	144
	Reverse: GACCATGTCCGTTCCCAAGT		
*GLUT4*	Forward: GGCGGATGCTATGGGTC	JQ343218.1	122
	Reverse: ACGGGTTTCAGGCACTTT		
*PCK1*	Forward: ACGCGCTTCCCGAATTCTCA	XM_004014441.1	130
	Reverse: TCCCCAACCTCTTTAGTGAC		
*PCK2*	Forward: AACAGCAGGGACTCATCCGAAA	XM_015096868.1	100
	Reverse: ATCACCGTCTTGCTCTCTACTCGT		
*PC*	Forward: TCGCACCATGTATGTCATCCC	NM_177946	187
	Reverse: AGGCTTTTTTAAAGGCAGAGGG		
*HK*	Forward: GCGGCTCTCTGATAAAACTCTGTTA	AM492192	139
	Reverse: TGAGCCATCGGGAATAGACCTTAC		
*IGF1R*	Forward: GGCTCAACCCAGGGAA	XM_018065947.1	164
	Reverse: CACTATCAACAGAACCGCAAT		
*IR*	Forward: GCCCTGGTGTCACTTTCC	XM_018051134.1	182
	Reverse: GCTGCCTTAGGTTCTGGTT		
*IRS1*	Forward: TGCCTGACCAGCAAGACCA	XM_018058864.1	187
	Reverse: CACCTGCATCCAGAACTCCC		
*PI3K*	Forward: CGAGCATTTCTGCTTTGGG	XM_018047551.1	152
	Reverse: GGTCTTGGAGGCATTGTTCTG		
*AKT*	Forward: CTAAGCAGCGGCTTGGTG	NM_001285750.1	165
	Reverse: GGTCAGGTGGCGTAATGGT		
*Atrogin1*	Forward: TAAACTTGTGCGATGCTAC	XM_005688865.3	167
	Reverse:TGTCATGTGCTCGGATT		
*MuRF1*	Forward:GAGCAAGGCAGGTGAAGG	XM_018058864.1	134
	Reverse: TGGCACGGCAAGATGAC		
*G6PC*	Forward:AATGTCATGTTGTGGTTGGGATTCT	EF062861	158
	Reverse: GCATTGTAGATGCTCTGGATGTGG		
*GAPDH*	Forward:GGGTCATCATCTCTGCACCT	HM043737.1	180
	Reverse: GGTCATAAGTCCCTCCACGA		
18S	Forward:GTGATGGGGATCGGGGATTG	[39]	172
	Reverse: GTAGCGACGGGCGGTGTGTA		

*GLUT1* Glucose transporter type 1, *GLUT2* Glucose transporter type 2, *SGLT1* Sodium glucose transporter type 1, *Na-K/ATPase* Sodium-potassium ATPase, *GLUT4* Glucose transporter type 4, *PCK1* Cytosolic form of phosphoenolpyruvate carboxykinase, *PCK2* Mitochondrial form of phosphoenolpyruvate carboxykinase, *PC* Pyruvate carboxylase, *HK* Hexokinase, *IGF1R* Insulin-like growth factor 1 receptor, *IR* Insulin receptor, *IRS1* Insulin receptor substrate1, *PI3K* Phosphoinositide 3-kinase, *AKT* AKT serine/threonine kinase 1, *Atrogin1* F-box protein 32, *MuRF1* Muscle RING-Finger protein 1, *G6PC* Glucose-6-phosphatase, *GAPDH* Glyceraldehyde-3-phosphate dehydrogenase

### Western blot analysis

A total 100 mg of frozen liver tissue was minced and homogenized in 1 mL of ice-cold RIPA buffer containing the protease inhibitor cocktail Complete EDTA-free (Roche, Penzberg, Germany). The homogenates were centrifuged at 12,235×*g* for 20 min at 4 °C and then the supernatant fraction was collected. After extraction, protein was diluted to the proper concentration with RIPA buffer. The protein concentration was determined using a BCA Protein Assay kit (Pierce, Rockford, IL, USA). Then, 50 μg of protein extract from each sample was separated by 10% SDS-PAGE, and the separated proteins were transferred onto nitrocellulose membranes (BioTrace; Pall Corp., New York, NY, USA). After transfer, membranes were blocked in blocking buffer with 5% skim milk for 2 h at room temperature. Next, the membranes were incubated overnight at 4 °C with the following primary antibodies: rabbit-anti-PCK1 (1:1,000; 16754–1-AP, Proteintech, Rosemont, IL, USA), mouse-anti-PCK2 (1:1,000; ab70359, Abcam, Cambridge, UK), and anti-TUBULINα (1:10,000; BS1699, Bioworld Technology, Saint Louis Park, MN, USA) in dilution buffer, which was consisted of 3% bovine serum albumin. After several washes in tris-buffered-saline with Tween, the membranes were incubated in dilution buffer for 2 h at room temperature with goat anti-rabbit HRP-conjugated or anti-mouse HRP-conjugated secondary antibodies (1:10,000; Bioworld Technology). Finally, the blot was washed and the proteins were detected by enhanced chemiluminescence using the LumiGlo substrate (Super Signal West Pico Trial Kit; Pierce) and the signals were recorded by an imaging System (Bio-Rad, Hercules, CA, USA) and analyzed with the Quantity One software (Bio-Rad).

### Statistical analysis

Data are presented as the mean ± SEM. The data were tested for normal distribution and analyzed by the Student’s unpaired t test or analysis of variance (ANOVA) using the SPSS software package (SPSS version 19.0 for Windows; SPSS Inc., Chicago, IL, USA). Data were considered statistically significant when *P* < 0.05.

## Results

### Dry matter intake (DMI) and body weight were decreased by Dex

**Fig. 1:**
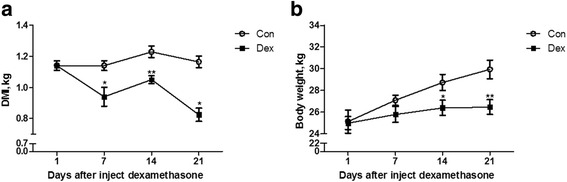
Changes in body weight and dry matter intake (DMI) in animals treated with dexamethasone (Dex). **a**, the dynamic changes of DMI during Dex treatment. **b**, the dynamic changes of body weight during the Dex treatment. Data are presented as the mean ± SEM. The data were analyzed by the independent-samples *t*-test using the Compare Means of the SPASS 19.0 software for Windows (StaSoft Inc., Tulsa, OK, USA). “*” indicates *P <* 0.05; “**” indicates *P <* 0.01

As shown in Fig. 1, the dry matter intake (DMI) (Fig. 1a) was significantly decreased after 7 d of Dex treatment compared with the control goats (*P* < 0.05). Also, the body weight (Fig. 1b) of Dex-treated goats was significantly decreased after 14 d compared with the control goats (*P* < 0.05).

### Plasma glucose and insulin concentrations were increased by Dex

**Fig. 2:**
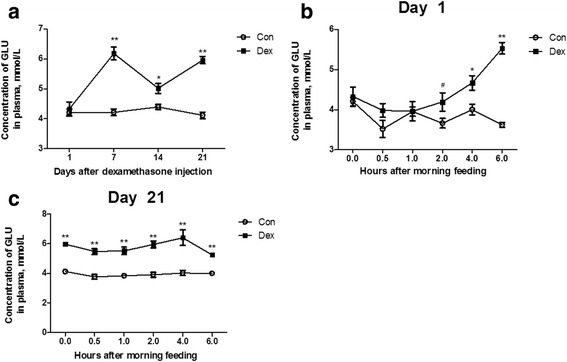
Chronic dexamethasone (Dex) treatment induced hyperglycemia in goat. **a**, the dynamic changes of plasma glucose during Dex treatment. **b**, the dynamic changes of plasma glucose after the first injection of Dex injection. **c**, the dynamic changes of plasma glucose after 21 d of Dex treatment. Data are presented as the mean ± SEM. The data were analyzed by the independent-samples *t*-test using the Compare Means of the SPASS 19.0 software for Windows (StaSoft Inc., Tulsa, OK, USA). “#” indicates 0.1 *< P <* 0.05; “*” indicates *P <* 0.05; “**” indicates *P <* 0.01

**Fig. 3:**
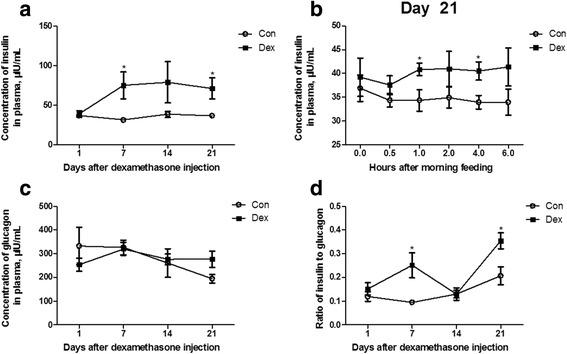
Chronic dexamethasone (Dex) treatment induced higher level of plasma insulin in goat. **a**, the dynamic changes of plasma insulin during Dex treatment. **b**, the dynamic changes of plasma insulin after 21 d of Dex treatment. **c**, the dynamic changes of plasma glucagon during Dex treatment. **d**, the dynamic changes of ratio of insulin to glucagon in plasma during Dex treatment. Data are presented as the mean ± SEM. The data were analyzed by the independent-samples *t*-test using the Compare Means of the SPASS 19.0 for software Windows (StaSoft Inc., Tulsa, OK, USA). “*” indicates *P <* 0.05

As shown in Fig. 1, the plasma glucose concentration (Fig. 2a) was significantly increased after 7 d of Dex treatment compared to the control goats (*P* < 0.05). The Dex treatment at all time points induced a gradual increase of blood glucose from the first injection onwards, and showed a statistical significance from the injection at 2 h (*P* < 0.05) (Fig. 2b), and after 21 d of injections, the blood glucose was higher in the Dex-treated goats than in the control goats at any time point (*P* < 0.01) (Fig. 2c). Consistently, the changes in the plasma insulin concentration by Dex showed the same pattern as those in the plasma glucose (Fig. 3a and b). The plasma glucagon concentration was not affected by Dex (Fig. 3c), but the ratio of insulin to glucagon was significantly increased at 7 d and 21 d in the Dex-treated animals compared with the control goats (Fig. 3d).

### Plasma cortisol concentrations were decreased by Dex

**Fig. 4:**
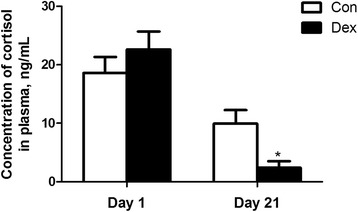
Chronic dexamethasone (Dex) exposure decreased plasma cortisol concentration in goat. Data are presented as the mean ± SEM. The data were analyzed by the independent-samples *t*-test using the Compare Means of the SPASS 19.0 software for Windows (StaSoft Inc., Tulsa, OK, USA). “*” indicates *P <* 0.05

As shown in Fig. 3, plasma cortisol concentrations was similar in the two groups before the experiment, but it was significantly decreased (*P* < 0.05) after 21 d of Dex treatment (Fig. 4), indicating that there is a strong negative feedback control of endogenous cortisol secretion.

### The glycogen content in liver and dorsal longissimus muscle

**Fig. 5:**
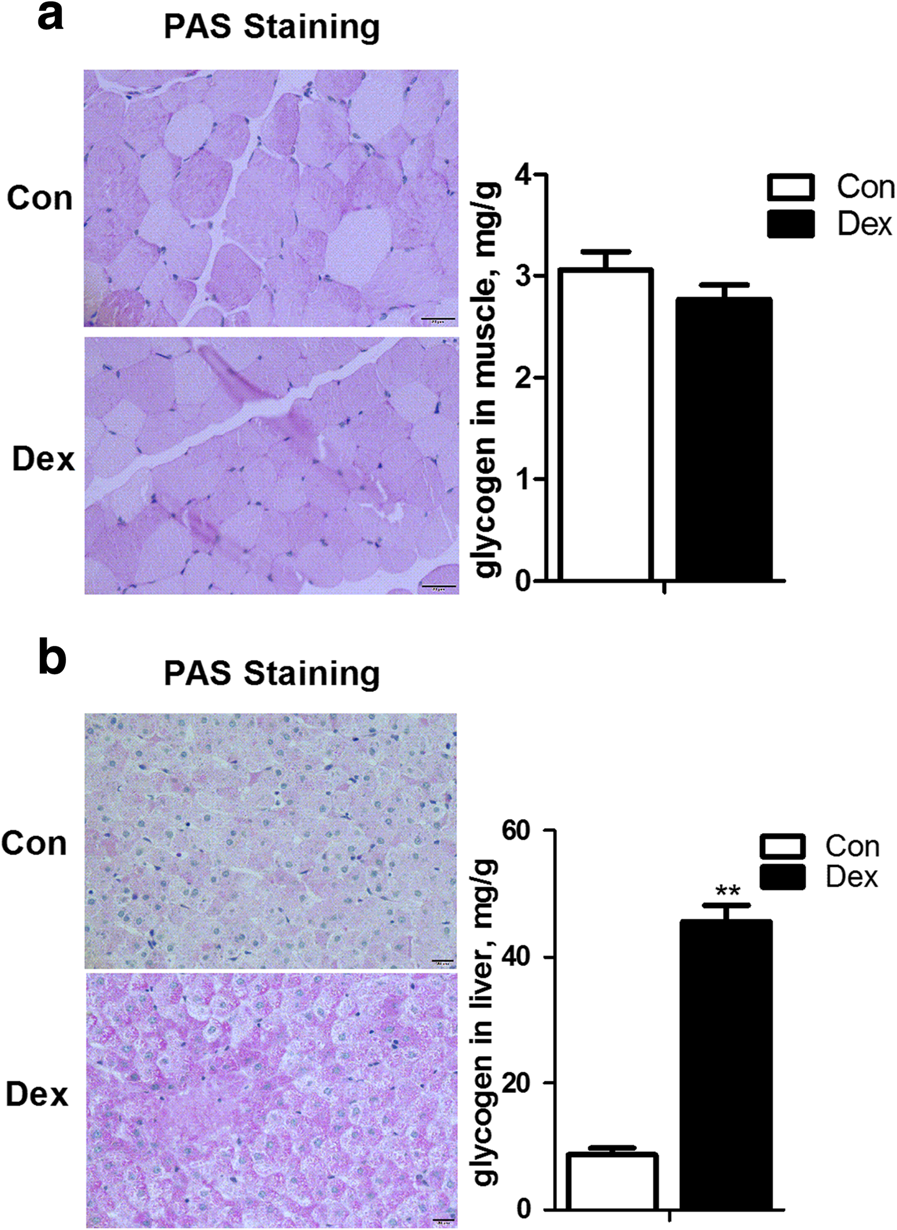
Effects of chronic treatment with dexamethasone (Dex) on glycogen deposition in the liver (**a**) and skeletal muscle (**b**). Data are presented as the mean ± SEM. The data were analyzed by the independent-samples *t*-test using the Compare Means of the SPASS 19.0 software for Windows (StaSoft Inc., Tulsa, OK, USA). “**” indicates *P <* 0.01

As shown in Fig. 4a, the content of glycogen in dorsal longissimus muscle was not changed by Dex treatment (Fig. 5a). On the other hand, hepatic glycogen content in Dex-treated goats was significantly increased compared with control goats (*P* < 0.01) (Fig. 5b).

### Expression of genes controlling glucose absorption in the duodenum

**Fig. 6:**
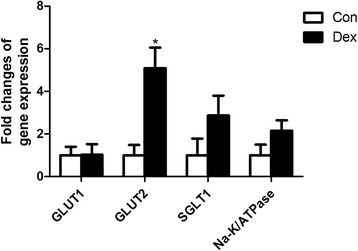
The expression of *GLUT1*, *GLUT2*, *SGLT1* and *Na-K/ATPase* in the duodenum of goats treated with dexamethasone (Dex). Data are presented as the mean ± SEM. The data were analyzed by the independent-samples *t*-test using the Compare Means of the SPASS 19.0 software for Windows (StaSoft Inc., Tulsa, OK, USA). “*” indicates *P <* 0.05

The expression of genes encoding proteins controlling glucose absorption in the duodenum was generally up-regulated by Dex. Among these genes, *GLUT2* mRNA expression was significantly increased by Dex (*P* < 0.05), indicating a higher glucose absorption ability by the small intestine in Dex-treated goats compared with the control counterparts (Fig. 6).

### The changes of glucose up-take and insulin signal pathway in dorsal longissimus muscle after Dex administration

**Fig. 7:**
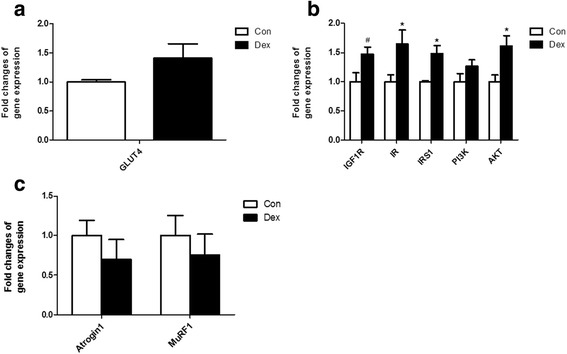
Relative gene expression in dorsal longissimus muscle. **a**, gene expression of *GLUT4* in skeletal muscle after Dex treatment. **b**, Expression of *IGF1R*, *IR*, *IRS1*, *PI3K* and *AKT* in goat muscle after Dex treatment. **c**, Expression of *Atrogin1* and *MuRF1* in skeletal muscle after Dex treatment. Data are presented as the mean ± SEM. The data were analyzed by the independent-samples *t*-test using the Compare Means of the SPASS 19.0 software for Windows (StaSoft Inc., Tulsa, OK, USA). “#” indicates 0.1 *< P <* 0.05; “*” indicates *P <* 0.05

The glucose transporter 4, encoded by *GLUT4* is the gate for controlling glucose uptake from blood into skeletal muscle. *GLUT4* expression was found to be increased in dorsal longissimus muscle in Dex-treated goats, but the increased did not reach statistical significance compared with that in the control (Fig. 7a). Genes associated with the insulin signaling pathway, including the insulin-like growth factor 1 receptor (*IGF1R*), insulin receptor (*IR*), insulin receptor substrate1 (*IRS1*), phosphoinositide 3-kinase (*PI3K*) and AKT serine/threonine kinase 1 (*AKT/PKB*) were also significantly up-regulated by Dex (Fig. 7b). Expression of *Atrogin1* and *MuRF1*, two markers of muscle atrophy, were unaltered by Dex at the transcriptional level (Fig. 7c).

### Gluconeogenesis was enhanced in liver by Dex

**Fig. 8:**
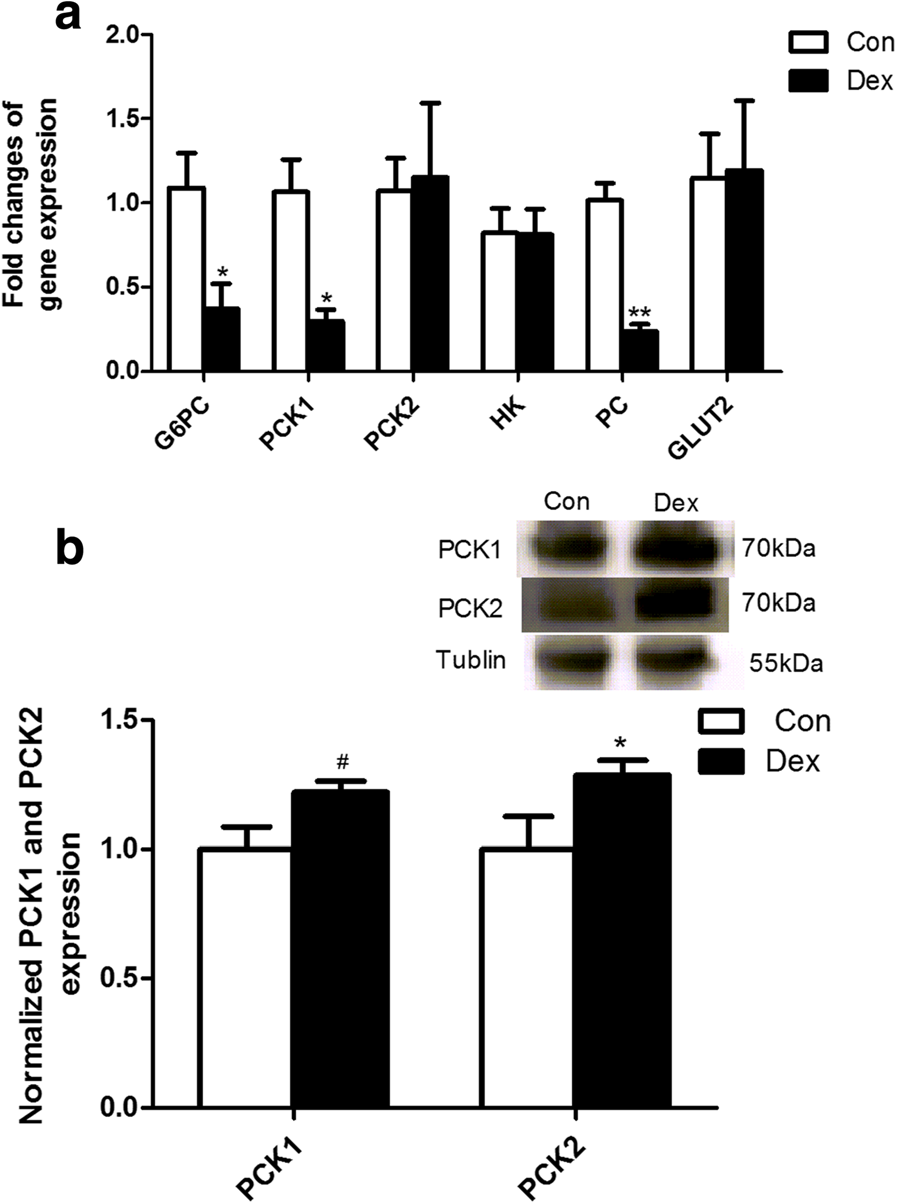
The expression of genes and proteins in the liver after Dex treatment. **a**, expression of genes involved in gluconeogenesis in the liver. **b**, expression of proteins related to gluconeogenesis in the liver. Data are presented as the mean ± SEM. The data were analyzed by the independent-samples *t*-test using the Compare Means of the SPASS 19.0 software for Windows (StaSoft Inc., Tulsa, OK, USA). “#” indicates 0.1 *< P <* 0.05; “*” indicates *P <* 0.05; “**” indicates *P <* 0.01

Real-time PCR analysis results showed that the expression of several key genes involved in hepatic gluconeogenesis including *G6PC*, *PCK1* and *PC* mRNA was markedly down-regulated by Dex (*P* < 0.05) (Fig. 8a). However, the protein level of PCK1 and the mitochondrial form of phosphoenolpyruvate carboxykinase (PCK2) protein detected by Western blot analysis were significantly increased in the liver of Dex-treated goats compared with the control (*P* < 0.05) (Fig. 8b), suggesting a post-transcriptional regulation mechanism.

## Discussion

In this study, we found that goats chronically exposed to Dex, a synthetic GC, exhibited a reduction in body weight, hyperglycemia and hepatic glycogen accumulation, which is consistent with similar findings by studies in other mammals. Dex treatment was found to reduce cortisol concentration in all mammals. It is well established that GCs exert feedback inhibition on their own secretion by blocking the effects of the corticotrophin releasing hormone on the pituitary gland [20, 21]. In this study, a significant decrease of endogenous cortisol secretion was observed in Dex-treated goats. These observations demonstrated that an experimental model of chronic GC administration to goats has been well established, and Dex-treated goats showed similar metabolic conditions as observed in other mammals.

The reduction in body weight was accompanied by a decrease in feeding and an increase in water consumption. The increase in water consumption may indicate a higher energy expenditure. Other studies indicate that Dex can reduce body weight without affecting food ingestion by increasing caloric expenditure [22]. GC-induced skeletal muscle atrophy through the decrease of the rate of protein synthesis and increase of the rate of protein breakdown has been reported [23]. The GC-stimulated atrophy is mediated through the increased expression of several Atrogenes (“genes involved in atrophy”), such as *Atrogin1*, and *MuRF*1 [23]. In this study, we did not find a significant difference in expression of these two genes in skeletal muscle of goats after chronic Dex treatment. Thus, the decrease in food intake and higher energy expenditure may lead to the lower body weight of Dex-treated goats. However, the occurrence of GC-induced muscle atrophy still cannot be excluded in this study, and needs further investigation.

The main metabolic effect of GCs is to positively regulate the supply of enough glucose into the circulation to fuel the brain and ensure survival of the organism under conditions of acute stress or starvation. These effects are critical during stress, which in the short run does not affect or even enhances glucose tolerance. However, chronic exposure to GC results in hyperglycemia and insulin resistance [24]. Moreover, other studies have reported that there was a marked increase in the ratio of insulin to glucagon in Dex-treated rats [25]. Our results are consistent with the available research in human and non-ruminant animals. In this study, we observed a transient increase in blood glucose, insulin and the insulin to glucagon ratio, which might be explained by an increase in the hepatic glycogen output resulting from the stimulation of gluconeogenesis and leads to an increase in peripheral resistance to insulin. Glucocorticoids exert tissue-specific effects on glucose metabolism. In the liver, GCs promote hepatic gluconeogenesis [26], however, they reduce glucose uptake and utilization in skeletal muscle and white adipose tissue [27], which coordinately contribute to hyperglycemia and peripheral insulin resistance. In ruminants, glucose is an important nutrient of metabolism [7], however, only a small amount of blood glucose is absorbed in the small intestine. Also, the source of glucose depends on hepatic gluconeogenesis by utilizing some precursors such as propionate produced from fermentation in rumen and glucogenic amino acids to meet their metabolic demand for glucose [9]. The conversion of propionate carbon to glucose is controlled by the abundance of *PCK2* and *G6PC* [28]. In non-ruminant mammals, Dex significantly increased gluconeogenic genes of *PCK1* and *G6PC* expression in hepatocytes via binding to the GCs responsive elements (GREs) of the *PCK1* and *G6PC* genes [29]. In this study, however, the expression of hepatic gluconeogenic genes mRNA were significantly decreased by chronic treatment with Dex, this discrepancy is explained by differences between the species. In contrast, the protein expression level of PCK1 and PCK2 was markedly increased in the liver of Dex-treated goats compared with normal control goats, suggesting the involvement of a post-transcriptional regulation by Dex, as well as a higher ability of hepatic gluconeogenesis in Dex-treated goats. Moreover, our results also showed a significant increase of hepatic glycogen accumulation in goats chronically exposed to Dex, which is consistent with research in other mammals [30, 31].

It’s well documented that GCs exert tissue-specific effects on glucose metabolism. Glucocorticoids inhibit glucose utilization by reducing both glucose uptake and oxidation in skeletal muscle and white adipose tissue, two major tissues involved in insulin-responsive glucose uptake [32, 33]. In the liver, several studies suggest that GC increases glycogen storage, whereas in skeletal muscle GC plays a permissive role for catecholamine-induced glycogenolysis or inhibit insulin-stimulated glycogen synthesis [34]. In contrast, Burke et al. [35] reported that when mice were chronically exposed to corticosterone, the expression of glycogen synthase 1 was greatly enhanced in muscle which was consistent with elevations in muscle glycogen storage. The present study revealed that Dex moderately increased muscular *GLUT4* mRNA expression and overall up-regulated the expression of glucogeogenic genes in goat skeletal muscle, however, the content of muscular glycogen content in goats was not found to be changed by Dex. One possible explanation for these findings is that the activities and protein abundances of muscular gluconeogenic enzymes are not coupled with their genes expression, as observed in liver of Dex-treated goats. Also, in this study, the expression of genes involved in the insulin signaling pathway was generally up-regulated by chronic exposure to Dex. Indeed, the results indicated a common insulin resistance induced by Dex in peripheral tissues, which was consistent with the significantly higher level of blood glucose and insulin in Dex-treated goats.

Unlike non-ruminant mammals, ruminants only absorb small amount of glucose in the small intestine. Here, a significant up-regulation of the expression of *GLUT2* mRNA was detected in Dex-treated goats. Some studies have reported that GCs increase the intestinal absorption of sugars in both young and mature rats [36]. Additionally, chronic stress was found to significantly increase the expression of *GLUT2* in the rat duodenum, which results in the increase of sugar up-take and contributes to the development of hyperglycemia [37]. Accordingly, it is reasonable to assume that the increase of *GLUT2* expression may facilitate sugar up-take in the duodenum epithelial membrane in Dex-treated goats, which may partially contribute to causing hyperglycemia. However, the mRNA expression of *SGLT1*, as well as *GLUT1* and *Na-K/ATPase* was not altered by chronic exposure of goats to Dex. In ruminants, whether the adverse effects of chronic exposure to GC on metabolic disorders can be reversed by steroid removal as observed in rats [35], still waits for further study.

## Conclusions

Our data collectively show that chronic Dex-treatment resulted in lower body weight, reduced hyperglycemia and higher hepatic glycogen accumulation in goats. According to the changes observed in hepatic genes expression, it is reasonable to assume that hyperglycemia caused by chronic exposure to Dex was mainly due to the activation of hepatic gluconeogenesis and insulin resistance.

## Abbreviations

AKT: AKT serine/threonine kinase 1
Atrogin1: F-box protein 32
G6PC: Glucose-6-phosphatase
GAPDH: Glyceraldehyde-3-phosphate dehydrogenase
GLUT1: Glucose transporter type 1
GLUT2: Glucose transporter type 2
GLUT4: Glucose transporter type 4
HK: Hexokinase
IGF1R: Insulin-like growth factor 1 receptor
IR: Insulin receptor
IRS1: Insulin receptor substrate1
MuRF1: Muscle RING-Finger protein 1
Na-K/ATPase: Sodium-potassium ATPase
PC: Pyruvate carboxylase
PCK1: Cytosolic form of phosphoenolpyruvate carboxykinase
PCK2: Mitochondrial form of phosphoenolpyruvate carboxykinase
PI3K: Phosphoinositide 3-kinase
SGLT1: Sodium glucose transporter type 1
